# Predicting long-term risk of sudden cardiac death with automatic computer-interpretations of electrocardiogram

**DOI:** 10.3389/fcvm.2024.1439069

**Published:** 2024-10-23

**Authors:** Minna Järvensivu-Koivunen, Antti Kallonen, Mark van Gils, Leo-Pekka Lyytikäinen, Juho Tynkkynen, Jussi Hernesniemi

**Affiliations:** ^1^ Faculty of Medicine and Health Technology, Tampere University, Tampere, Finland; ^2^ Tays Heart Hospital, Tampere University Hospital, Tampere, Finland; ^3^ Department of Radiology, Tampere University Hospital, Tampere, Finland; ^4^ Finnish Cardiovascular Research Center Tampere, Tampere University, Tampere, Finland

**Keywords:** acute coronary syndrome, sudden cardiac death, machine learning, computer interpretation, electrocardiogram

## Abstract

**Background:**

Computer-interpreted electrocardiogram (CIE) data is provided by almost all commercial software used to capture and store digital electrocardiograms. CIE is widely available, inexpensive, and accurate. We tested the potential of CIE in long-term sudden cardiac death (SCD) risk prediction.

**Methods:**

This is a retrospective of 8,568 consecutive patients treated for acute coronary syndrome. The primary endpoint was five-year occurrence of SCDs or equivalent events (SCDs aborted by successful resuscitation or adequate ICD therapy). CIE statements were extracted from summary statements and measurements made by the GE Muse 12SL algorithm from ECGs taken during admission. Three supervised machine learning algorithms (logistic regression, extreme gradient boosting, and random forest) were then used for analysis to find risk features using a random 70/30% split for discovery and validation cohorts.

**Results:**

Five-year SCD occurrence rate was 3.3% (*n* = 287). Regardless of the used ML algorithm, the most significant risk ECG risk features detected by the CIE included known risk features such as QRS duration and factors associated with QRS duration, heart rate–corrected QT time (QTc), and the presence of premature ventricular contractions (PVCs). Risk score formed by using most significant CIE features associated with the risk of SCD despite adjusting for any clinical risk factor (including left ventricular ejection fraction). Sensitivity of CIE data to correctly identify patients with high risk of SCD (over 10% 5-year risk of SCD) was usually low, but specificity and negative prediction value reached up to 96.9% and 97.3% when selecting only the most significant features identified by logistic regression modeling (*p*-value threshold <0.01 for accepting features in the model). Overall, CIE data showed a modest overall performance for identifying high risk individuals with area under the receiver operating characteristic curve values ranging between 0.652 and 0.693 (highest for extreme gradient boosting and lowest for logistic regression).

**Conclusion:**

This proof-of-concept study shows that automatic interpretation of ECG identifies previously validated risk features for SCD.

## Introduction

Sudden cardiac death (SCD) is a significant cause of death in the general population and even more so in patients with known coronary artery disease (CAD) (1, 2). Most SCD victims are patients with mildly reduced or normal left ventricular ejection fraction (LVEF), who are therefore not eligible for primary prevention with an implantable cardioverter-defibrillator (3, 4). During the last few decades, multiple plausible electrocardiographic (ECG) risk factors for SCD depicting autonomic abnormalities have been identified, such as heart rate, signs of myocardial scarring, and signs of abnormal ventricular depolarization or repolarization (5–12). Several potential ECG-based composite risk scores have also been developed (13–15). These results seem promising, but they have not led to actual advances in clinical practice, perhaps due to the lack of replicability and the low sensitivity. In previous ECG risk score studies, usually incorporating 4–6 partly overlapping risk markers, the only consistently emerging risk factor for SCD has been left ventricular hypertrophy (LVH) (13–15), and even then the SCD risk associated with LVH has been approximately 2.2–2.5-fold in discovery cohorts but usually greatly reduced in multivariable analyses and in validation studies (13–15). The replicability issue is partly influenced by the heterogeneity of the used risk markers, although they usually depict the same phenomena and the small number of cases available in discovery cohorts of prospective trials and the even smaller number of cases in replication cohorts (14, 15). The lack of other large cohort studies with high-quality endpoint data for SCD and access to standardized ECG data is a significant challenge (13, 14, 16).

One potential solution to the replicability problem in the research on SCD could be to use computerized interpretation of the ECG (CIE) to provide standardized phenotype data. There are even promising results of CIE outperforming experienced physicians (17). GE-Marquette 12SL ECG analysis (GE Healthcare, Milwaukee, WI, USA) is a standardized computerized interpretation program that is used globally (18). Our aim was to evaluate the prognostic value and feasibility to use data of basic measurements and statement combinations by GE-Marquette in the prediction of SCD.

## Methods

### Study design and cohort

This study is based on a retrospective analysis of the real-life data of patients treated in a tertiary center, collected in a single research database (MADDEC study) (19). The scientific monitoring committee of Pirkanmaa Hospital District approved the study. The study complies with the Declaration of Helsinki ethical principles for medical research.

Between January 2007 and December 2018, 10,314 consecutive patients underwent coronary angiography for ACS in Tampere Heart Hospital (a part of Tampere University Hospital). The Heart Hospital is the sole specialized health care provider in cardiologic emergencies in a region of over 0.5 million inhabitants, and all patients undergoing invasive diagnostics within this region are treated in the study center. ACS was defined as an ST elevation MI (STEMI), non-ST elevation MI (NSTEMI), or unstable angina pectoris (UAP), according to ESC guidelines (20). In Tampere Heart Hospital, less than 10% of patients with suspected ACS do not undergo coronary angiography, usually due to a poor estimated prognosis and overall condition (21).

**Figure 1:**
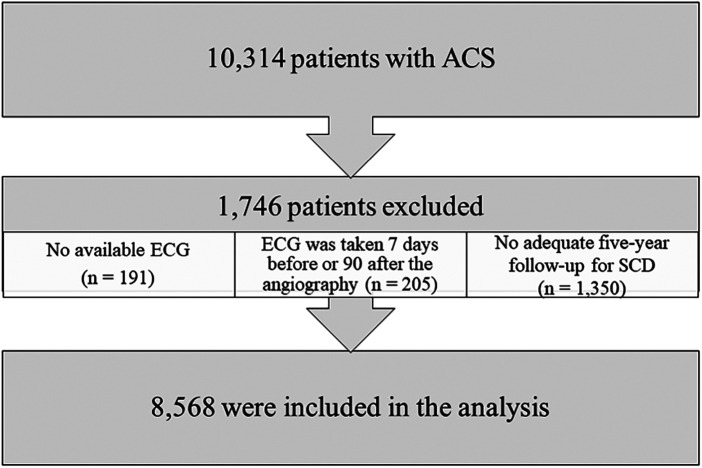
Patient flowchart.

Out of all 10,314 consecutive patients undergoing angiography for ACS, patients with no electronic ECG available (*n* = 191) and those for whom the ECG was recorded more than 7 days prior to, or over 90 days after, the angiography (*n* = 205) were excluded. Finally, as follow-up time was limited to 5 years (last follow-up date Dec 31, 2021), patients without adequate five-year follow-up data for SCD were excluded (*n* = 1,350; this criterion applies to patients treated between 2017 and 2018 who were alive at the end of the follow-up). After exclusions, 8,568 patients were available for analysis. The majority (*n* = 8,239, 96.2%) of the ECGs were recorded on the same day, as or within 1 week after, the angiography. A flowchart of the patient selection is presented in Figure 1.

### Data collection

The MADDEC (Mass Data in Detection and Prevention of Serious Adverse Events in Cardiovascular Disease) database was used to obtain laboratory results and information on patients’ medical history. The database combines written patient record data from specialized healthcare with electronic health records, such as laboratory results, diagnoses, body measurements, and ECGs, and with the KARDIO registry, which contains information on diagnostic procedures and treatments (19). Information on deaths was obtained from Statistics Finland, patient records, and death certificates with written descriptions of the manner of death (see below for a more detailed description of SCD endpoint adjudication).

**Figure 2:**
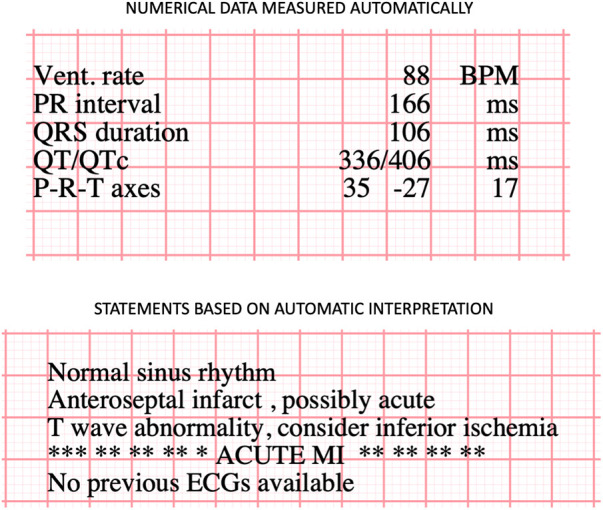
Example of computer interpreted ECG statement by using GE Marquette 12SL data.

All available standard 12-channel ECGs recorded after angiography, or before if an ECG was not available after angiography (*n* = 9), were considered. The ECGs were recorded as a part of the normal clinical workflow by a trained laboratory technician or a nurse with standard commercially available GE ECG recording devices, and the recordings were stored in the MUSE database hosted by Fimlab Laboratories. The ECGs were interpretated using the 12SL GE Marquette program, and the ECGs were stored in pdf format, from which the interpretations were extracted by using an R program (package pdftools) (22). The text format interpretations included statements and numerical measurement of the present ECG selected for analysis (Figure 2 for example). Possible statements comparing the present ECG to the previous one was not included (this feature is available if there are previous ECGs available for interpretation in the database). Before testing the associations between ECG interpretations and SCD, potentially clinically relevant subgroups of features were further merged to form additional ECG variables. A full list of ECG statements and the subgroups formed *a priori* following clinical rationale is presented in Supplementary Table S1.

### Endpoint definitions

The primary endpoint is a composite SCD event—denoting true SCDs and cases where a patient would most likely have died an SCD without intervention, i.e., accurate ICD therapy for ventricular arrhythmia (VA) or successful resuscitation (with or without anoxic brain damage)—occurring within five years of ACS. In order to identify only risk markers specific to SCD, patients who remained alive at five years or who died within five years of other causes were regarded as controls. A death was classified as an SCD if it was presumably of cardiac etiology and occurred within 1 h of the onset of symptoms, or if the patient was found dead within 24 h of being asymptomatic. The classification was based on the AHA/ACC/HRS and ESC guidelines (3, 23). If a hospitalized patient had prolonged cardiac symptoms; if the patient had a deteriorating clinical condition, severe dementia, or was in palliative care; or if the description of the manner of death was vague, the death was not classified as an SCD. Successfully resuscitated patients were identified by screening written patient records of Tampere University Hospital, where all resuscitated patients who survive until reaching the hospital are treated, and suspected cases were further assessed by an in-depth review of all written patient records. Pacemaker events of patients with an ICD were screened to identify accurate ICD therapies. ICD therapy readings were then confirmed individually by reviewing the written patient records detailing the pacemaker ECG data and the classification, therapy, and description of the event. In the case of multiple endpoint episodes, the first one was chosen for the analysis. For sensitivity analysis, patients with ICD devices (either received before ACS or anytime during the five-year observation period) were additionally excluded from the study. Endpoint data were collected until the 31st of December 2021.

### Statistical methods

The *t*-test was used to test differences between groups for continuous variables, while Pearson’s *X*^2^ test was applied for categorical variables. The overall dimensionality of ECG features was reduced with principal component analysis (PCA) based on Eigenvalues (>1 with no fixed limit for components) using IBM SPSS Statistics software (v. 29.0.1.0). A threshold for the number of components to maintain was obtained to explain 95% of the ECG data variance. These principal components (PC) were then used in the logit model adjusted for sex and age to calculate their association with SCD. PCA was performed using the whole data set without train/validation split.

To screen for potential CIE-based risk markers and to validate their predictive value, the population was divided into independent training and validation samples with a random division of 70% (training) and 30% (validation). This split was chosen based on our data size. Based on training data, a five-year risk of SCD was calculated for all patients. Three different supervised machine learning (ML) risk prediction models were built to predict SCD: logistic regression analysis (LR), random forest (RF) and Extreme Gradient Boost (XGB) (24–26).

The logistic regression model was constructed by filtering the most significant ECG features, first by testing which features were associated with SCD risk, with a nominal *p*-value of 0.05 or less. These nominally significant ECG features were then introduced to the model by a forward stepwise algorithm using a conservative *p*-value of 0.01 for entry and removal from the model. Random forest is an ensemble learning method that operates by constructing a multitude of decision trees at training time and outputting the class that is the mode of the classes of the individual trees for classification tasks. In this case, two classes represented the SCD endpoints. The optimization of the random forest parameters was conducted using a grid search algorithm. This method involves evaluating a model across a range of algorithm parameters specified in a grid to identify the combination that optimizes the model’s performance. Specifically, the parameters optimized for the random forest model included the max_depth of the tree, tested at 10, 60, and 100; the min_samples_leaf, which is the minimum number of samples required at a leaf node, tested at 1, 2, and 4; and the min_samples_split, or the minimum number of samples necessary to split an internal node, tested at 2, 5, and 10. During the grid search, all possible combinations of the specified parameter values were evaluated, and the best combination was retained. The best parameters for each model were then selected based on their performance on the development set. This process was repeated for each of the specified models. Following the grid search, the optimal parameters for the random forest were determined to be a max_depth of 10, min_samples_leaf of 4, and min_samples_split of 10. Parameter optimization, training, and data visualization were performed using Python version 3.10.12 with the packages sklearn, pandas, and matplotlib. The Python codings are introduced specifically in Supplementary Table S2. Extreme gradient boosting was performed with the R package xgboost, and hyperparameters for extreme gradient boosting were optimized with the package ParBayesianOptimization The optimized hyperparameters were eta (range 0–1), gamma (0–20), max depth (1–20), min child weight (2–30), subsample (0.1–1), and max delta steps (0–20). The tuning function used a 10-fold cross-validated extreme gradient boosting model, max rounds of 75, and early stop rounds 20. Both unscaled and scaled models were performed (scale pos weight = 30) to evaluate performance after prediction. Also, unscaled risk was used to test the 10% SCD threshold. The tuning was performed with seven initial starting points, in 100 iterations and at 5 times per epoch. Optimized hyperparameters (non-scaled values for SCDv5: eta = 0.4614819, gamma = 2.108328, max_depth = 1, min_child_weight = 2.496067, subsample = 1, max_delta_step = 20; the number of rounds in the best iteration was also extracted) were used in the model training, and the model performance was finally tested in the validation set.

Model calibration was tested using the Hosmer–Lemeshow test with ten risk level/stratification groups. Only XGB model showed suboptimal fit among the very highest risk categories (*p* = 0.01 comparing predicted risk and occurred events, see data supplement). Feature importance was assessed by feature importance algorithms in each ML; Gini (analyzing the change in the model’s prediction error after permuting the feature) in RF, Gain (training loss reduction gained when using a feature for splitting) in XGB and regression coefficient in LR.

Additionally, the clinical stratification value (performance) of the risk scores was tested by analyzing their predictive value over *a priori* defined, clinically meaningful thresholds for SCD risk in the validation sample using an area under the receiver operating characteristic (AUROC) curve. For the purpose of this study, a 10% five-year risk was defined as a threshold of interest, after which an ICD is likely to be indicated. This threshold was selected as it corresponds roughly with the 2%–3% yearly SCD event rate in control populations of trials testing the efficacy of ICD devices (27–34). It is also clearly above the actionable limit for ICD implantation in hypertrophic cardiomyopathy. The sensitivity, specificity, positive and negative prediction values were reported.

## Results

### Characteristics

At baseline, the mean age of the entire population was 68.3 years (±11.8 SD), and 67.3% (*n* = 6,671) of the patients were male. There were no significant differences between training and validation sets in baseline characteristics or in the occurrence of SCDs (Table 1).

**Table 1:** Baseline characteristics of patients undergoing coronary angiography for acute coronary syndrome in Tampere Heart Hospital between 2007 and 2018.

	All patients (*n* = 8,568)	Training set (*n* = 6,012)	Validation set (*n* = 2,556)	*P*-value^a^
Age	68.4 (11.9)	68.4 (11.8)	68.4 (12.1)	0.890
Men,% (*n*)	67.3% (5,768)	67.2% (4,043)	67.5% (1,725)	0.829
Acute coronary syndrome type				0.259
Unstable angina	19.2% (1,646)	19.5% (1,175)	18.4% (471)	
Non-ST elevation myocardial infarction	45.2% (3,877)	44.7% (2,688)	46.5% (1,189)	
ST elevation myocardial infarction	35.5% (3,045)	35.7% (2,149)	35.1% (896)	
Coronary artery disease severity				0.359
No flow-limiting occlusions	11.1% (1,103)	11.0% (764)	11.4% (339)	
1-vessel disease	38.6% (1,145)	39.4% (2,735)	38.6% (1,145)	
2-vessel disease	25.9% (770)	26.9% (1,871)	25.9% (770)	
3-vessel disease	24.1% (716)	22.7% (1,578)	24.1% (716)	
Killip classifications for heart failure				0.699
I	77.3% (6,606)	77.6% (4,655)	76.5% (1,951)	
II	14.2% (1,213)	14.1% (843)	14.5% (370)	
III	6.9% (556)	6.3% (381)	6.9% (175)	
IV	2.2% (55)	2.0% (121)	2.2% (55)	
Previous history of myocardial infarction	17.8% (1,529)	17.7% (1,066)	18.1% (463)	0.674
Diabetes	25.5% (2,177)	25.7% (1,539)	25.0% (638)	0.560
Peripheral artery disease	8.0% (681)	7.8% (467)	8.4% (214)	0.345
Hypertension	60.1% (5,135)	60.1% (3,607)	59.9% (1,528)	0.889
History of cancer (any)	8.4% (708)	8.7% (516)	7.6% (192)	0.101
Valvular heart disease (any)	7.3% (627)	7.3% (436)	7.5% (191)	0.720
History of chronic or acute kidney disease	12.5% (1,072)	12.4% (744)	12.9% (328)	0.545
Dyslipidemia	57.1% (4,873)	57.1% (3,422)	57.0% (1,451)	0.935
History of stroke	8.3% (713)	8.2% (492)	8.6% (221)	0.478
Median creatinine, umol/l (interquartile range)	77 (66–92)	77 (66–91)	77 (67–92)	0.238
Mean hemoglobin, mg/dl (SD)	129.3 (16.0)	129.6 (16.0)	128.8 (16.0)	0.040
Mean left ventricular ejection fraction,% (SD)^b^	51.2 (11.9)	51.2 (11.8)	51.2 (12.0)	0.812
SCD occurrence rate	3.3% (287)	3.5% (209)	3.3% (78)	0.317
Overall mortality rate	23.6% (2,026)	41.3% (2,483)	40.5% (3,519)	0.508

^a^ Comparison between values in the statistical analysis training set and validation set.

^b^ Data available for 75.1% of all patients in the study population (*n* = 6,435/8,568).

### Rate and incidence of events and association between SCD and traditional risk factors

During the five-year follow-up period, 2,026 patients died (23.6%) and 287 SCDs or equivalent events were recorded, 80.1% (*n* = 230) of which occurred in patients without ICDs. Based on these events, the five-year occurrence rate of SCDs or equivalent events was 3.3% and the SCD occurrence rate among patients without ICDs was 2.7%. The unadjusted and adjusted associations (in the form of odds ratios) between SCD and traditional risk factors in the entire study population are presented in Table 2.

**Table 2:** Association between traditional risk factors and sudden cardiac death among patients undergoing coronary angiography for acute coronary syndrome in Tampere Heart Hospital between 2007 and 2018.

	Univariable odds ratio (95% CI)	Multivariable odds ratio (95% CI)	Multivariable odds ratio (95% CI)^a^
Age	1.07 (0.97–1.19)	0.92 (0.82–1.03)	0.92 (0.80–1.06)
Men,% (*n*)	1.56 (1.18–2.04)	1.59 (1.12–2.26)	1.39 (0.97–1.99)
Acute coronary syndrome type			
Unstable angina	Reference	Reference	Reference
Non-ST elevation MI	1.97 (1.36–2.88)	1.63 (1.01–2.41)	1.42 (0.93–2.19)
ST elevation MI	1.58 (1.06–2.34)	1.44 (0.95–2.19)	0.94 (0.57–1.54)
Coronary Artery Disease Severity			
No flow-limiting occlusions	Reference	Reference	Reference
1-vessel disease	2.33 (1.24–4.38)	2.05 (1.01–4.18)	2.18 (1.03–4.63)
2-vessel disease	3.70 (1.97–6.95)	2.52 (1.23–5.17)	2.45 (1.14–5.25)
3-vessel disease	4.28 (2.29–8.03)	2.43 (1.17–5.02)	2.41 (1.12–5.19)
Killip classification			
I	Reference	Reference	Reference
II	2.41 (1.81–3.20)	1.96 (1.39–2.75)	1.54 (1.07–2.21)
III	3.25 (2.29–4.60)	2.24 (1.44–3.50)	1.56 (0.97–2.52)
IV	1.61 (0.74–3.48)	1.24 (0.49–3.15)	0.97 (0.38–2.56)
Previous history of MI	2.52 (1.97–3.24)	2.18 (1.60–2.96)	1.59 (1.12–2.27)
Dyslipidemia	1.04 (0.82–1.32)	0.74 (0.55–1.00)	0.80 (0.58–1.10)
Valvular heart disease	1.39 (0.93–2.07)	0.85 (0.52–1.41)	0.83 (0.50–1.39)
Cancer (any)	1.05 (0.69–1.61)	0.89 (0.56–1.40)	0.93 (0.58–1.48)
Chronic obstructive pulmonary disease	2.23 (1.56–3.19)	1.57 (1.04–2.38)	1.48 (0.97–2.27)
History of stroke	1.51 (1.04–2.17)	0.79 (0.49–1.28)	0.77 (0.47–1.26)
Diabetes	1.99 (1.56–2.54)	1.55 (1.15–2.11)	1.62 (1.16–2.26)
Peripheral artery disease	2.39 (1.73–3.30)	1.42 (0.96–2.11)	1.36 (0.90–2.05)
Hypertension	1.16 (0.91–1.48)	0.95 (0.70–1.30)	1.01 (0.73–1.39)
History of kidney disease	2.07 (1.56–2.75)	0.96 (0.63–1.47)	0.90 (0.58–1.40)
Serum creatinine	1.24 (1.70–1.32)	1.16 (1.06–1.27)	1.20 (1.09–1.32)
Left ventricular ejection fraction	0.54 (0.47–0.61)	-	0.62 (0.53–0.73)

^a^ Model tested in a subsample (75%) of patients with LVEF data available (*n* = 6,435/8,568).

### Principal component analysis

**Figure 3:**
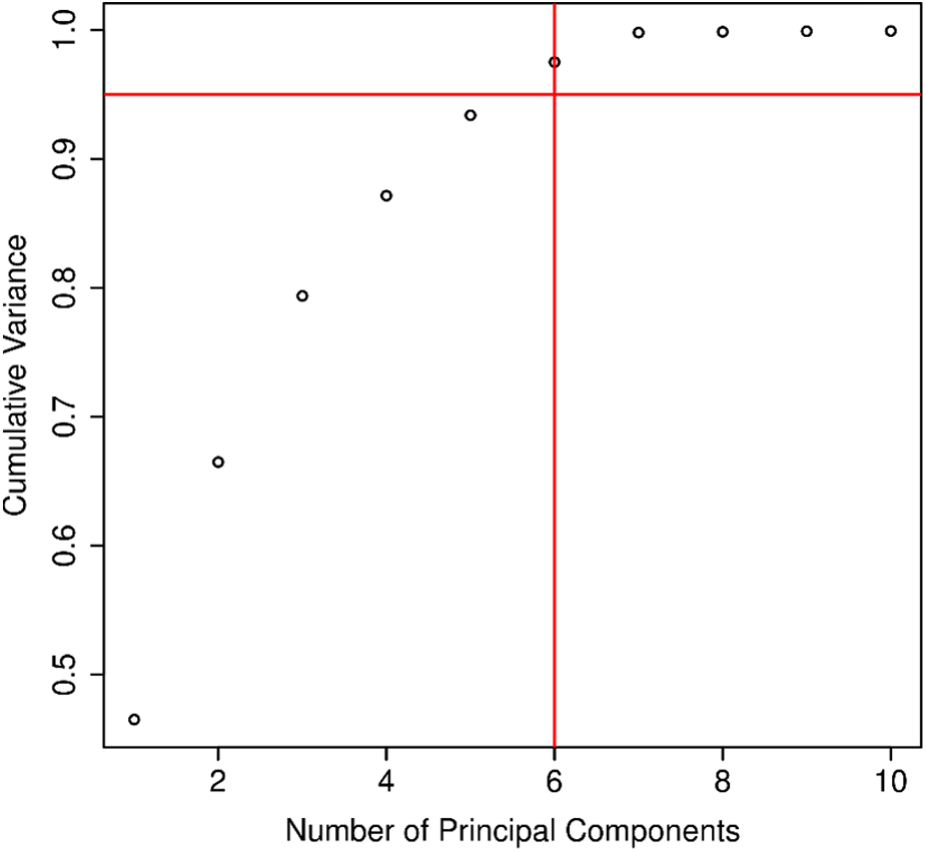
The number of principal components and their relation to the explained variance in the electrocardiographic (ECG) data extracted from 12-channel ECGs by Marquette 12SL software in patients treated for acute coronary syndrome (*n* = 8,568).

The overall variance in the data obtained from CIE statements was analyzed with principal component analysis (PCA). The six first PCs explained over 95% of the variance and were introduced to a logit model adjusted for age and sex (Figure 3). Together, these six principal components reached an AUROC value of 0.642, predicting SCD in the entire study population.

### Machine learning models and SCD prediction

**Figure 4:**
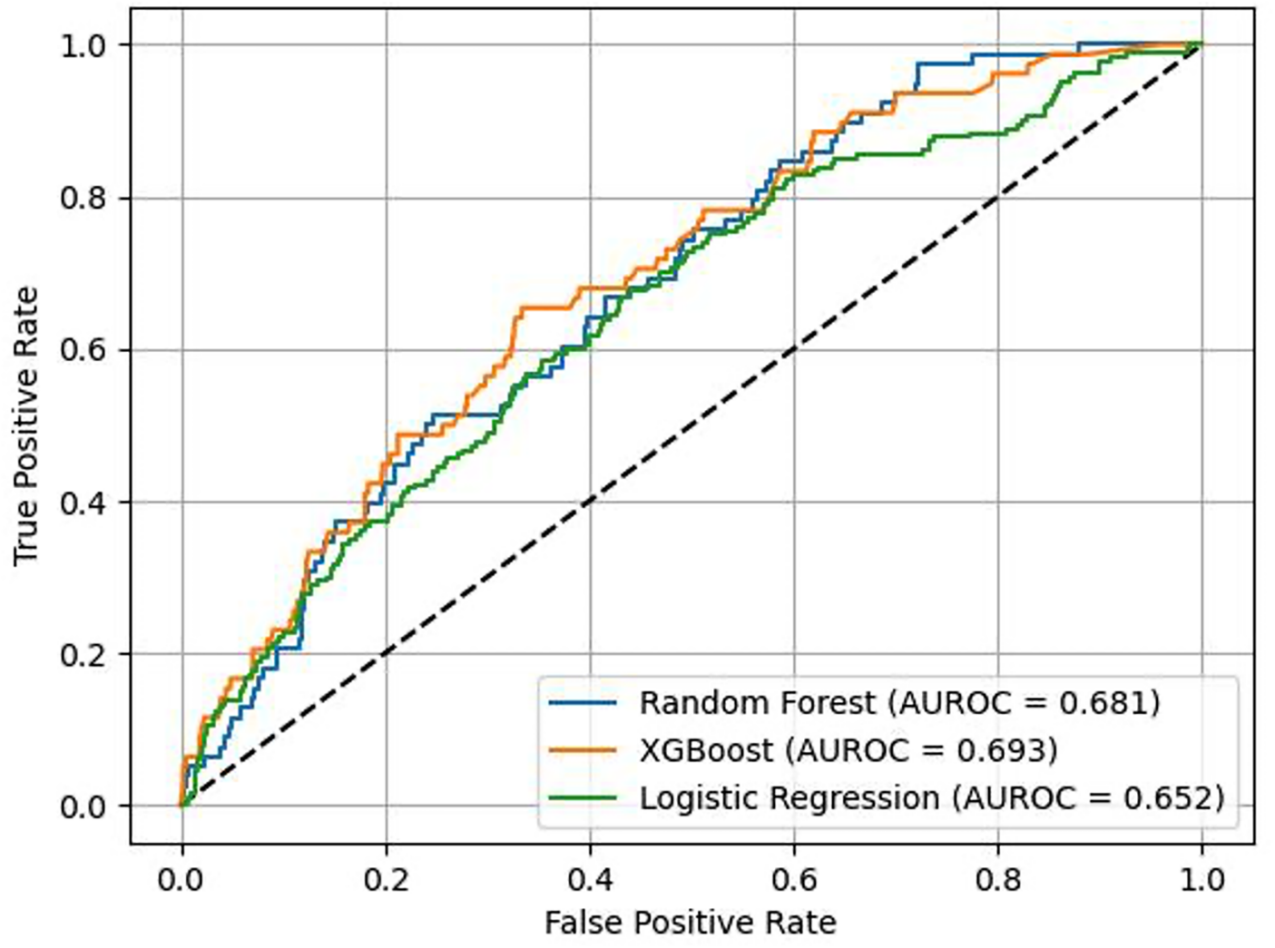
The overall performance, as reflected in an ROC plot, of different machine learning algorithms using computer-interpreted electrocardiogram statements for the prediction of sudden cardiac death or equivalent event within five years of an acute coronary syndrome event.

**Figure 5:**
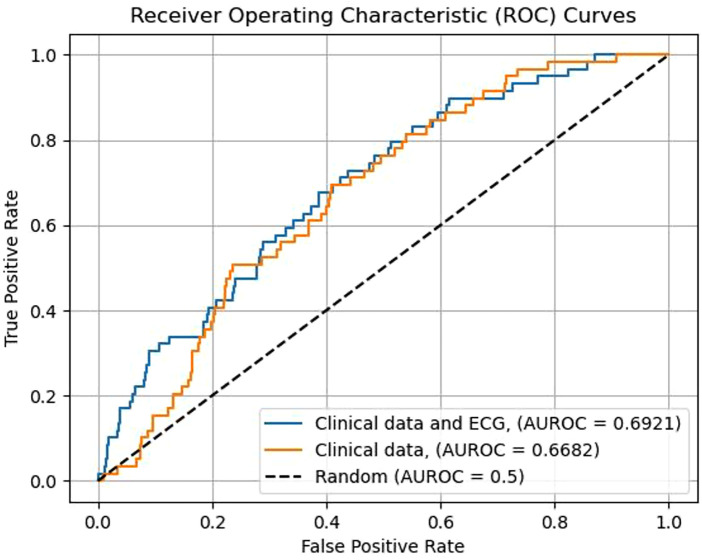
Incremental predictive value of ECG parameters added to information obtained from traditional risk factors in predicting the five-year occurrence of all SCD events. Models constructed with the random forest machine learning algorithm. Clinical variables used in the model: age, serum creatinine value, hemoglobin value, Killip classification for heart failure, dyslipidemia, hypertension, diabetes, prevalent cancer, valvular heart disease, peripheral artery disease, chronic obstructive pulmonary disease, history of kidney failure, patient sex.

**Figure 6:**
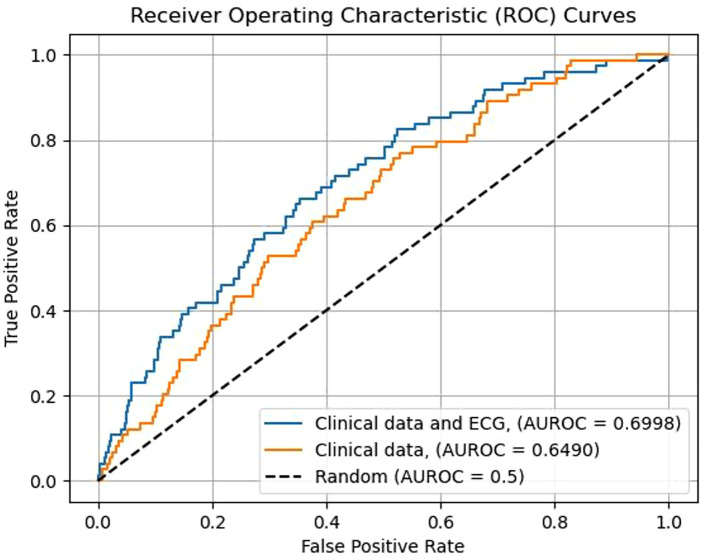
Incremental predictive value of ECG parameters added to information obtained from traditional risk factors in predicting the five-year occurrence SCDs among patients not eligible for an intra-cardiac defibrillator device. Models constructed with the random forest machine learning algorithm. Clinical variables used in the model: age, serum creatinine value, hemoglobin value, Killip classification for heart failure, dyslipidemia, hypertension, diabetes, prevalent cancer, valvular heart disease, peripheral artery disease, chronic obstructive pulmonary disease, history of kidney failure, patient sex.

The performance of ML risk prediction models is presented in Figure 4. In brief, the highest AUROC values were attributed to the XGB (0.693) and RF models (0.681) and the lowest to LR (0.652). For reference, using clinical data (age, sex, creatinine, hemoglobin, Killip class, dyslipidemia, hypertension, diabetes, cancer, valvular heart disease, peripheral arterial disease, chronic obstructive pulmonary disease, and kidney disease as variables) and the RF classifier, the resulting AUROC was 0.668. Adding ECG data to this RF prediction model increased the AUROC to 0.692, indicating that ECG variables have incremental predictive value (Figure 5). The same observation was made if the analysis was repeated after excluding patients with ICDs (Figure 6). Additionally, when the association between LR-based standard risk prediction metric (range of predicted risk values between 0 and 1) and SCD was analyzed before and after adjusting the model for significant clinical risk factors, the association remained statistically significant and similar indicating that the association between ECG features and SCD is independent of traditional risk factors (including left ventricular ejection fraction) (Table 3).

**Table 3:** The association between ECG statement–based continuous risk score (by logistic regression analysis) and SCD with or without adjusting for traditional risk factors and LVEF in the validation sample. The odds ratio (OR) estimate corresponds to a one standard deviation increase in the ECG risk score. Adjusting variables for Model 3 and Model 4 were selected by taking all significant variables associating with the risk of SCD in the training population.

	OR for SCD	*p*-value	OR for SCD (no ICDs)^a^	*p*-value
Unadjusted Model	1.39 (1.23–1.57)	<0.001	1.38 (1.21–1.58)	<0.001
Model 2	1.35 (1.19–1.53)	<0.001	1.33 (1.15–1.52)	<0.001
Model 3	1.27 (1.11–1.46)	<0.001	1.28 (1.10–1.49)	0.001
Model 4	1.26 (1.08–1.47)	0.003	1.31 (1.10–1.56)	0.002
Model 5	1.32 (1.16–1.51)	<0.001	1.33 (1.15–1.54)	<0.001

Model 2 adjusted for age and sex; Model 3 adjusted for age, sex, diabetes, history of myocardial infarction, CAD severity, Killip classification, prevalent chronic obstructive pulmonary disease, and serum creatinine; Model 4 adjusted for age, sex, diabetes, history of myocardial infarction, CAD severity, serum creatinine, and left ventricular ejection fraction; Model 5 adjusted for left ventricular ejection fraction.

^a^ All patients who received an implantable cardioverter defibrillator before an acute coronary syndrome event or within five years of the event are excluded.

### Variable importance

The most important ECG features in the RF model and XGB are listed by rank in Table 4, which also presents the variables (features) selected by LR with their corresponding odds ratios (calculated from the validation dataset). Regardless of the applied ML algorithm, the same common features were highlighted. These included previously identified risk factors for SCD, such as QRS duration or factors associated with QRS duration (presence of non-specific intraventricular conduction disorders or incomplete left bundle branch block), heart rate–corrected QT time (QTc), and the presence of premature ventricular contractions (PVCs). T wave axis and R wave axis were also top-ranked by the XGB and RF algorithms (Table 4).

**Table 4:** The top features in random forest, extreme gradient boosting and logistic regression models in predicting sudden cardiac death.

	Metric for importance
Extreme Gradient Boosting	Gain
QRS duration	0.216
QTc duration	0.121
Lateral ST segment depression (isolated)	0.111
Premature ventricular contractions	0.108
T wave axis	0.106
Random Forest	Gini
QRS duration	0.0708
QTc duration	0.0645
T wave axis (missing values replaced by mean)	0.0608
T wave axis	0.0596
R wave axis	0.0577
Logistic Regression	Regression coefficient^a^
QRS duration (for one SD increase)	1.19 (0.95–1.48)
Premature ventricular contractions	2.10 (1.12–3.93)
Non-specific intraventricular conduction disorder/block	2.72 (0.97–7.61)
Lateral ST segment depression	1.93 (0.94–3.87)
Incomplete left bundle branch block	5.57 (2.06–15.04)

^a^ Variables not ranked by metric of importance and coefficients presented as odds ratios in a multivariable model with all features simultaneously in the same model.

### The applicability of different ML models using CIE statements for identifying patients at high risk of SCD

The predictive value of CIE statements by different ML models in identifying patients at a high (10%) five-year risk of SCD is presented in Table 5. LR identified only a small fraction of the population (3.3%) at high risk, with subsequently low sensitivity (14.1%) and PPV (12.6%), but with high specificity (96.9%) and negative predictive value (97.3%). XGB performed very similarly in identifying high-risk individuals (Table 5). Using the RF algorithm, a ten-fold number of patients compared to LR analysis were identified to have a high risk (37.8%), with higher sensitivity (61.5%) but lower specificity (63.0%) (Table 5).

**Table 5:** The ability of the studied risk prediction algorithms to identify patients at a high risk of sudden cardiac death (10% or higher five-year SCD risk).

	Specificity	Sensitivity	NPV	PPV	Prevalence
Endpoint: all SCDs					
Logistic regression	96.9%	14.1%	97.3%	12.6%	3.3%
Random forest	63.0%	61.5%	98.1%	5.0%	37.8%
Extreme gradient boosting	98.1%	9.0%	97.2%	13.1%	2.1%
Endpoint: SCDs among patients with no ICDs					
Logistic regression	96.4%	14.3%	97.7%	9.4%	2.5%
Random forest	95.8%	12.7%	97.7%	7.3%	4.4%
Extreme gradient boosting	99.1%	3.2%	97.5%	8.7%	0.9%

Abbreviations: NPV, negative predictive value; PPV, positive predictive value; ICD, implantable cardioverter-defibrillator.

## Discussion

In this study, we used a large retrospective cohort of consecutive patients treated for ACS with high-quality endpoint data for SCD to evaluate the prognostic value of CIE in the prediction of SCD. According to our observations, CIE can be used to identify patients at high risk for SCD, but the overall performance is modest. ML algorithms, such as random forest and XGB perform better than standard regression modeling when measuring AUROC values across the entire risk spectrum. However, in identifying high-risk patients for clinical purposes, a conventional and conservatively built regression model performs adequately, although the sensitivity of the model is low (14%). According to our sensitivity analysis, CIE-based risk prediction also works to identify patients at risk of SCD outside of ICD indications, and CIE parameters are independent of traditional risk factors.

There are no similar previous studies to which can compare our results to. Although internally validated, our results require outside validation. However, using different ML methods, we observed that QRS duration and related features, such as nonspecific intraventricular conduction delay (NIVCD) and incomplete left bundle branch block (LBBB), are all significantly associated with the risk of SCD. Although these results are based on fully automated interpretation, they align very well with previous observations (6, 7, 35–37). Not surprisingly, QTc time was also a major component in the SCD prediction models. Furthermore, the presence of premature ventricular contractions also seemed to associate with SCD (38). In contrast to many studies, elevated heart rate was not predictive of SCD event possibly due to high risk of competing events in patients with heart failure (39).

Complex ML algorithms have gained interest in recent medical research. We also tested different ML algorithms, given the complexity of the CIE-produced data. As a rule, ML models must balance between interpretability and performance, where a more highly performing model must sacrifice interpretability and vice versa (40). Model suitability is dependent on data size, quality, and complexity, as well as the goals and preferences of the study (40, 41). The best-known and perhaps the simplest ML algorithms, linear and logistic regression, perform well in the absence of complex relationships and are often easier to use and interpret than modern ML algorithms (41, 42). Decision trees are tree-like structured algorithms that represent decisions and possible consequences (41, 43). Methods such as the “boosting” or “bagging” of multiple decision trees are used in more complex algorithms, such as the RF and XGB used in our study (41, 43, 44). In our data, XGB and RF algorithms produced the highest AUROC values. However, as evidenced by the principal component analysis, the overall complexity of our data set was not high, despite integrating several hundred parameters (mostly with low frequency), and there was very little difference in the performance of different ML methods.

There have been several attempts to develop ECG-based SCD risk prediction scores using conventionally interpreted (and often manually or semi-automatically measured) ECG. The risk score developed in the Oregon Sudden Death Study (Oregon SUDS) was validated in the prospective Atherosclerosis Risk in Communities (ARIC) study, in which individuals from the general population with extremely high scores (4–6 positive risk markers, prevalence 1.1%) had an approximately 2%–2.5% five-year incidence of SCD (13). The risk score developed in the prospective PREDETERMINE study (patients with established CAD but no indication for ICD device) was validated in the ARTEMIS study, in which CAD patients with high risk scores (prevalence of 10%) were observed to have a roughly 5.2% five-year cumulative incidence of sudden arrhythmic death (14). Unfortunately, we lack the data of several components used in these ECG scores and are unable to compare the results directly. In our study, with all available data from automated statements, we were able to identify approximately 3.3% of the population at high risk (over 10% five-year risk) of SCD using a regression analysis–based model. This model had low sensitivity (only capturing 14% of all SCD cases). Still, primary prevention based on this risk scoring would target patients with an event rate comparable to the control populations of trials testing the efficacy of ICDs in patients with a low LVEF (27–33). Compared to traditional ECG risk markers, the results obtained using CIE can be rapidly repeatable everywhere with corresponding outcome data because it uses (manufacturer-dependent) algorithms for fast and mostly accurate evaluation of recorded ECGs given the sufficient signal quality (45).

Potential SCD risk stratification tools are also based on clinical data (46). However, their applicability is usually subject to heterogeneity as regards the baseline risk and the definitions of the different components of the risk scores. Recently, a composite risk score (VFRisk) for SCA was developed using clinical, echocardiographic, and ECG-based parameters in a case–control setting (the Oregon SUDS). Subsequently, four of the thirteen components were ECG parameters. The VFRisk successfully discriminates SCA cases from controls with an internally and externally validated AUROC value of 0.782 (47). While these results are promising, the problem is that, in contrast to many ECG parameters, traditional clinical risk factors for SCD (or for SCA) are also risk factors for deaths due to other causes and, therefore, their applicability in identifying patients who would benefit from targeted SCD prevention with an ICD device is ambiguous. All possible risk scores should be validated in a clearly defined population while controlling for mortality due to other causes during follow-up. Compared to more general risk factor–based risk stratification tools, ECG is more likely to provide more cardiac-specific information that can stratify the population at high risk of specifically SCD but at a low risk of death due to other causes. For example, many of the major components of the risk score developed in the ARIC cohort, such as age, serum albumin, and renal function, are also associated with a high risk of death due to other causes (46, 48).

### Limitations

As our cohort only included patients with previous ACS, it is not generalizable to patients with no previous heart conditions. In particular, the observations concerning individual potential risk factors should be considered cautiously because many of the features are only seen in patients after ACS (and with significant coronary artery disease). Also, the cohort is from a limited geographical area in Finland, comprising mainly Caucasian individuals, which limits the generalizability to geographically and ethnically different populations. Furthermore, CIE algorithms differ by manufacturer, and clinical interpretation is still required if the actual predictive value of specific ECG features needs to be verified (45). In addition, updates to the algorithms over time were not considered in our study, and they are subject to change over time. However, this means that using the same software version for all recordings would probably only improve the predictive performance of CIE when the heterogeneity in the data is reduced. Furthermore, we did not have full five-year follow-up data for subjects treated in years 2017 and 2018 and still alive at the end of the follow-up period (December 31st of 2021) and thus they were not included in our analyses (13% of all patients). This exclusion of control patients from later years could lead to some bias in our analyses. However, as these excluded patients had similar age and sex distribution when compared to patients included to control population in our analyses, it is likely that the current control population represents adequately those not at risk of SCD.

Our data are based on ECG recordings made at the time of ACS, and only on automated interpretations of recorded ECGs (i.e., not on the raw signal). For this reason, the full depth/potential of standardized 12-channel ECG data is probably under-represented. With longitudinal data and a more complete data matrix, the predictive value of ECG may be substantially better and more likely the low problem of low sensitivity of ECG based models can be addressed better.

### Strengths

The strengths of the present study include the reliable endpoint definitions for SCD, which are based on a full-disclosure review of all patient records and accounts of the circumstances leading to death. The autopsy rate of cardiac deaths in the present population was 29% between 2007 and 2019, and, overall, Finland has one of the highest autopsy rates in Northern Europe (49). Similarly, our results are based on a population of ACS patients with very minimal selection bias because the study center is the sole service provider for invasive diagnostics and care in the geographical region of Pirkanmaa, Finland (50). In the study center, less than 10% of all patients treated for myocardial infarction do not undergo an invasive evaluation due to poor overall functional capacity, severe neurodegenerative disability, or prognosis (21). However, in these patients, autopsy rates are lower and the prevention of SCD is usually no longer considered a clinical imperative. Additionally, one clear advantage of the present study is that, when searching for ECG risk markers for SCD, we were able to compare SCD cases to the pooled control group of patients who were still alive at the end of the five-year follow-up and patients who died of other causes within that time span. While this approach can lead to missing some risk factors associated with both a higher risk of SCD and mortality due to other cardiovascular causes, the approach provides the best opportunity to screen for SCD-specific risk markers and the utility of ECG to reveal SCD specific prognostic factors.

## Conclusion

The results of this proof-of-concept study show that CIE statements can be used to stratify patients at high risk of SCD, but the overall performance is modest. CIE could perhaps be used to guide primary SCD prevention. However, using ECG data from only one recording after ACS can only identify a very small proportion of patients at high risk without significant sacrifices in terms of specificity and positive prediction value. Given the feasibility of CIE, these results can be replicated in any cohort with ECG data in electronic format. This method also allows for rapid screening for SCD-specific ECG risk markers from serial recordings, which can increase the sensitivity of risk prediction. Future research should be directed to using more detailed parameters in risk model development with the clear intention to find patients at particularly high risk of SCD, despite the possibly low sensitivity of such models, so that ECG data could be used to direct clinical trials aiming at primary prevention of SCD.

## Data Availability

The data analyzed in this study is subject to the following licenses/restrictions: The data is available in fully anonymised form for research purpose upon resonable request pending the approval of the MADDEC study steering committee. Requests to access these datasets should be directed to jussi.hernesniemi@sydansairaala.fi.
