# Supplementary material for: Predicting long-term risk of sudden cardiac death with automatic computer-interpretations of electrocardiogram
Source: Front Cardiovasc Med. 2024 Oct 23;11:1439069. doi: 10.3389/fcvm.2024.1439069 (PMC11537987; doi:10.3389/fcvm.2024.1439069)
Supplement: Supplementary file 1 [file Datasheet1.docx]

Supplementary Material

Järvensivu-Koivunen et al**: Predicting sudden cardiac death with computer-interpreted electrocardiographic data**

**Description of ML models**

The logistic regression model was constructed by filtering the most significant ECG features, first by testing which features were associated with SCD risk, with a nominal p-value of 0.05 or less. These nominally significant ECG features were then introduced to the model by a forward stepwise algorithm using a conservative p-value of 0.01 for entry and removal from the model.

Random forest is an ensemble learning method that operates by constructing a multitude of decision trees at training time and outputting the class that is the mode of the classes of the individual trees for classification tasks. In this case, two classes represented the SCD endpoints. The optimization of the random forest parameters was conducted using a grid search algorithm. This method involves evaluating a model across a range of algorithm parameters specified in a grid to identify the combination that optimizes the model's performance. Specifically, the parameters optimized for the random forest model included the max_depth of the tree, tested at 10, 60, and 100; the min_samples_leaf, which is the minimum number of samples required at a leaf node, tested at 1, 2, and 4; and the min_samples_split, or the minimum number of samples necessary to split an internal node, tested at 2, 5, and 10. During the grid search, all possible combinations of the specified parameter values were evaluated, and the best combination was retained. The best parameters for each model were then selected based on their performance on the development set. This process was repeated for each of the specified models. Following the grid search, the optimal parameters for the random forest were determined to be a max_depth of 10, min_samples_leaf of 4, and min_samples_split of 10. Parameter optimization, training, and data visualization were performed using Python version 3.10.12 with the packages sklearn, pandas, and matplotlib.

Extreme gradient boosting was performed with the R package xgboost, and hyperparameters for extreme gradient boosting were optimized with the package ParBayesianOptimization (33,34). The optimized hyperparameters were eta (range 0–1), gamma (0–20), max depth (1–20), min child weight (2–30), subsample (0.1–1), and max delta steps (0-20). The tuning function used a 10-fold cross-validated extreme gradient boosting model, max rounds of 75, and early stop rounds 20. Both unscaled and scaled models were performed (scale pos weight = 30) to evaluate performance after prediction. Also, unscaled risk was used to test the 10% SCD threshold. The tuning was performed with seven initial starting points, in 100 iterations and at 5 times per epoch. Optimized hyperparameters (non-scaled values for SCDv5: eta = 0.4614819, gamma = 2.108328, max_depth=1, min_child_weight= 2.496067, subsample=1, max_delta_step = 20; the number of rounds in the best iteration was also extracted) were used in the model training, and the model performance was finally tested in the validation set.

Supplementary Figure 1. Example of computer interpreted ECG statement by using GE Marquette 12SL data.

Supplementary Figure 2. Model calibration for XGB model showing statistical significant (p=0.01) difference between predicted risk (red dots) and occurred events (black dots) due to variation in highest end of the risk continuum


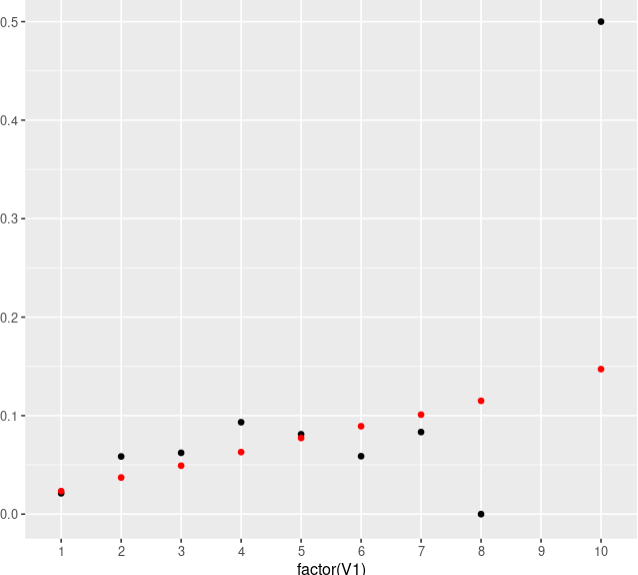


Supplementary Table 1. The list of all Computer identified electrocardiogram features and additional composite variable generated based on them. Composite features were comprised by combining information from ECG statements.

| FEATURE NAME / ECG STATEMENT | MEAN (SD) /  prevalence  (for categories with positive findings) |
| --- | --- |
|  |  |
| vent_rate | 70.7 (15.7) |
| pr_interval | 172.9 (31.9) |
| qrs | 100.4 (20.8) |
| p | 47.6 (25.8) |
| r | 12.5 (44.2) |
| t | 70.1 (81.4) |
| qt | 424.3 (49.2) |
| qtc | 453 (41.8) |
| p_MRM (Missing valies replaced with mean) | 47.5 (24.3) |
| t_MRM (Missing valies replaced with mean) | 70.1 (81.1) |
| pr_inteval_MRM (Missing valies replaced with mean) | 172.9 (29.8) |
| ACUTEMI | .075 |
| ACUTEMISTEMI | .002 |
| IncreasedRSratioinV1considerearlytransitionorposterio | .002 |
| Normalsinusrhythm | .470 |
| Atrialfibrillation | .078 |
| Atrialflutter | .007 |
| Junctional_rhythm | .007 |
| Ectopic_rhythm | .007 |
| Pacemaker_Ventricular_or_Atrial | .015 |
| Wide_QRS_rhythm | .004 |
| Undeterminedrhythm | .005 |
| AV_dissociation | .004 |
| Prematureatrialcomplexes | .030 |
| sinusarrhythmia | .047 |
| Prematuresupraventricularcomplexes | .013 |
| Leftventricularhypertrophy | .056 |
| MinimalvoltagecriteriaforLVHmaybenormalvariant_A | .004 |
| ModeratevoltagecriteriaforLVHmaybenormalvariant_A | .002 |
| Bifascicularblock | .011 |
| Leftanteriorfascicularblock | .031 |
| Leftbundlebranchblock | .028 |
| RVH | .001 |
| Rightbundlebranchblock | .054 |
| Leftposteriorfascicularblock | .002 |
| Incompleteleftbundlebranchblock | .010 |
| Incompleterightbundlebranchblock | .002 |
| Nonspecificintraventricularconductiondelay | .002 |
| Nonspecificintraventricularconductionblock | .017 |
| LowvoltageQRS | .050 |
| PossibleLeftatrialenlargement | .003 |
| Pulmonarydiseasepattern | .000 |
| repolarizationabnormality | .045 |
| VentricularpreexcitationWPWpatterntypeA | .000 |
| VentricularpreexcitationWPWpatterntypeB | .000 |
| Indeterminateaxis | .001 |
| AbnormalQRSTangleconsiderprimaryTwaveabnormality | .000 |
| Considerrightventricularinvolvementinacuteinferiorinfarct | .012 |
| Rightatrialenlargement | .002 |
| Rightventricularhypertrophy | .001 |
| Voltagecriteriaforleftventricularhypertrophy | .015 |
| AbnormalECG | .676 |
| BorderlineECG | .009 |
| Poordataqualityinterpretationmaybeadverselyaffected | .157 |
| Suspectarmleadreversalinterpretationassumesnoreversal | .002 |
| shortPR | .005 |
| SinusrhythmwithshortPR | .003 |
| WideQRSrhythm | .004 |
| Electronicatrialpacemaker | .002 |
| Prematureventricularcomplexes | .058 |
| Septalinfarct | .047 |
| Septalinfarctageundetermined | .021 |
| Septalinfarctnew | .000 |
| Septalinfarctpossiblyacute | .000 |
| Anteroseptalinfarct | .063 |
| Anteroseptalinfarctageundetermined | .014 |
| Anteroseptalinfarctnew | .000 |
| Anteroseptalinfarctpossiblyacute | .006 |
| Anteriorinfarct | .028 |
| Anteriorinfarctageundetermined | .010 |
| Anteriorinfarctnew | .000 |
| Anteriorinfarctpossiblyacute | .004 |
| Anteriorinjurypattern | .002 |
| Anterolateralinfarct | .021 |
| Anterolateralinfarctageundetermined | .005 |
| Anterolateralinfarctnew | .000 |
| Anterolateralinfarctpossiblyacute | .002 |
| Anterolateralinjurypattern | .000 |
| Inferiorinfarct | .169 |
| Inferiorinfarctageundetermined | .064 |
| Inferiorinfarctnew | .002 |
| Inferiorinfarctpossiblyacute | .005 |
| Inferiorposteriorinfarct | .013 |
| Inferiorinjurypattern | .002 |
| Inferiorposteriorinfarctageundetermined | .004 |
| Inferiorposteriorinfarctpossiblyacute | .001 |
| Inferolateralinjurypattern | .001 |
| Lateralinfarct | .008 |
| Lateralinfarctageundetermined | .004 |
| Lateralinfarctnew | .000 |
| Lateralinfarctpossiblyacute | .000 |
| Lateralinjurypattern | .010 |
| Posteriorinfarct | .001 |
| STelevationconsideranteriorinjuryoracuteinfarct | .003 |
| STelevationconsideranterolateralinjuryoracuteinfarct | .002 |
| STelevationconsiderinferolateralinjuryoracuteinfarct | .001 |
| MarkedSTabnormalitypossibleanteriorsubendocardialinjury | .005 |
| MarkedSTabnormalitypossiblelateralsubendocardialinjury | .007 |
| MarkedSTabnormalitypossibleinferolateralsubendocardialinj | .001 |
| MarkedSTabnormalitypossibleanterolateralsubendocardialinj | .004 |
| MarkedSTabnormalitypossibleinferiorsubendocardialinjury | .004 |
| STTwaveabnormalityconsideranterolateralischemia | .044 |
| STTwaveabnormalityconsiderinferolateralischemia | .019 |
| STTwaveabnormalityconsideranteriorischemia | .011 |
| STTwaveabnormalityconsiderinferiorischemia | .023 |
| STTwaveabnormalityconsiderlateralischemia | .061 |
| STTwaveabnormalityconsideranteriorischemiaordigitalis | .001 |
| STTwaveabnormalityconsideranterolateralischemiaordigi | .005 |
| STTwaveabnormalityconsiderinferiorischemiaordigitalis | .004 |
| STTwaveabnormalityconsiderinferolateralischemiaordigi | .003 |
| STTwaveabnormalityconsiderlateralischemiaordigitalis | .007 |
| STMarkedTwaveabnormalityconsiderlateralischemia | .001 |
| STMarkedTwaveabnormalityconsideranterolateralischemia | .037 |
| STMarkedTwaveabnormalityconsideranteriorischemia | .001 |
| STMarkedTwaveabnormalityconsiderinferolateralischemia | .000 |
| STMarkedTwaveabnormalityconsiderinferiorischemia_A | .001 |
| Twaveabnormalityconsideranteriorischemia | .034 |
| Twaveabnormalityconsiderinferiorischemia | .045 |
| Twaveabnormalityconsideranterolateralischemia | .131 |
| Twaveabnormalityconsiderinferolateralischemia | .030 |
| Twaveabnormalityconsiderlateralischemia | .144 |
| Twaveabnormalityconsideranteriorischemiaordigitaliseffe | .002 |
| Twaveabnormalityconsideranterolateralischemiaordigitalis | .007 |
| Twaveabnormalityconsiderinferolateralischemiaordigitalis | .004 |
| Twaveabnormalityconsiderlateralischemiaordigitaliseffec | .012 |
| NonspecificSTandTwaveabnormality | .031 |
| NonspecificSTandTwaveabnormalityprobablydigitaliseffec | .004 |
| NonspecificSTabnormality | .016 |
| NonspecificTwaveabnormality | .049 |
| NonspecificTwaveabnormalityprobablydigitaliseffect | .003 |
| MarkedSTabnormalitypossibleanteroseptalsubendocardialinju | .002 |
| MarkedTwaveabnormalityconsideranterolateralischemia | .052 |
| Markedsinusbradycardiawith1stdegreeAVblockwithFusionc | .000 |
| Markedsinusbradycardiawith1stdegreeAVblockwithoccasion | .000 |
| Markedsinusbradycardiawith1stdegreeAVblockwithPrematur | .000 |
| Markedsinusbradycardiawithmarkedsinusarrhythmiawithshort | .000 |
| MarkedsinusbradycardiawithPrematuresupraventricularcomplex | .000 |
| VentricularpacedrhythmwithfrequentPrematureventricularcom | .000 |
| WideQRSrhythmwithfrequentPrematureventricularcomplexes | .000 |
| WideQRStachycardia | .000 |
| @1stdegreeAVblock | .098 |
| @21AVconduction | .000 |
| @2nddegreeAVblock | .002 |
| @41AVconduction | .000 |
| Markedsinusbradycardia | .038 |
| considerinferolateralischemia | .032 |
| Sinusbradycardia | .198 |
| Sinusrhythm | .149 |
| Sinusatrialcapture | .000 |
| variableAVblock | .005 |
| Ventricularpacedrhythm | .000 |
| digitaliseffect | .043 |
| AcceleratedJunctionalrhythm | .003 |
| AVdissociation | .002 |
| Electronicventricularpacemaker | .008 |
| Junctionalbradycardia | .000 |
| Junctionalrhythm | .006 |
| Atrialfibrillationwithprematureventricularoraberrantlycon | .015 |
| AtrialflutterwithvariableAVblock | .005 |
| AVsequentialordualchamberelectronicpacemaker | .001 |
| Demandpacemakerinterpretationisbasedonintrinsicrhythm | .004 |
| LeftventricularhypertrophywithQRSwidening | .018 |
| LeftventricularhypertrophywithQRSwideningandrepolarizatio | .009 |
| Leftaxisdeviation | .116 |
| Leftventricularhypertrophywithrepolarizationabnormality | .036 |
| JunctionalbradycardiawithoccasionalPrematureventricularcom | .000 |
| JunctionalrhythmwithfrequentPrematureventricularcomplexes | .000 |
| Markedsinusbradycardiawith1stdegreeAVblock | .006 |
| Normalsinusrhythmwithsinusarrhythmia | .015 |
| pacemaker | .015 |
| ProlongedQT | .152 |
| Sinusbradycardiawith1stdegreeAVblock | .027 |
| Sinusbradycardiawith1stdegreeAVblockwithFusioncomplexe | .000 |
| SinusbradycardiawithoccasionalPrematureventricularcomplexe | .004 |
| SinusbradycardiawithPrematureatrialcomplexes | .004 |
| Sinusrhythmwith1stdegreeAVblock | .057 |
| Sinusrhythmwith1stdegreeAVblockwithoccasionalandconse | .001 |
| Sinusrhythmwith2nddegreeAVblockwithoccasionalPremature | .000 |
| SinusrhythmwithAVdissociationandJunctionalrhythm | .000 |
| SinusrhythmwithFusioncomplexes | .004 |
| SinusrhythmwithoccasionalPrematureventricularcomplexesand | .001 |
| SinusrhythmwithshortPRwithPrematuresupraventricularcompl | .000 |
| SinusrhythmwithsinusarrhythmiawithoccasionalPrematureven | .001 |
| UnusualPaxispossibleectopicatrialrhythm | .004 |
| UnusualPaxispossibleectopicatrialtachycardia | .001 |
| AcceleratedJunctionalrhythmwithoccasionalandconsecutivePr | .000 |
| Atrialfibrillationwithslowventricularresponse | .001 |
| Sinusbradycardiawith1stdegreeAVblockwithoccasionalPrem | .001 |
| SinusbradycardiawithfrequentandconsecutivePrematureventri | .000 |
| SinusbradycardiawithFusioncomplexes | .000 |
| Sinusbradycardiawithmarkedsinusarrhythmia | .003 |
| Sinusbradycardiawithmarkedsinusarrhythmiawith1stdegreeA | .001 |
| SinusbradycardiawithoccasionalandconsecutivePrematurevent | .000 |
| SinusbradycardiawithPrematuresupraventricularcomplexes | .001 |
| SinusbradycardiawithPrematureventricularcomplexesorFusion | .000 |
| Sinusbradycardiawithsinusarrhythmia | .009 |
| Sinusbradycardiawithsinusarrhythmiawith1stdegreeAVbloc | .001 |
| Sinusrhythmwith1stdegreeAVblockwithfrequentPrematurev | .000 |
| Sinusrhythmwith1stdegreeAVblockwithoccasionalPremature | .003 |
| Sinusrhythmwith2nddegreeAVblock | .001 |
| SinusrhythmwithfrequentPrematureventricularcomplexes | .005 |
| Sinusrhythmwithmarkedsinusarrhythmiawith1stdegreeAVbl | .002 |
| SinusrhythmwithoccasionalPrematureventricularcomplexes | .025 |
| SinusrhythmwithPrematureatrialcomplexes | .017 |
| SinusrhythmwithPrematuresupraventricularcomplexes | .009 |
| SinusrhythmwithPrematuresupraventricularcomplexeswithocca | .001 |
| Sinusrhythmwithsinusarrhythmia | .007 |
| Sinusrhythmwithsinusarrhythmiawith1stdegreeAVblock | .002 |
| Sinustachycardia | .028 |
| Sinustachycardiawith2nddegreeAVblock | .001 |
| SinustachycardiawithfrequentPrematureventricularcomplexes | .000 |
| SinustachycardiawithFusioncomplexes | .001 |
| SinustachycardiawithPrematureatrialcomplexes | .002 |
| UnusualPaxispossibleectopicatrialbradycardia | .002 |
| WideQRStachycardiahasreplacedSinusrhythm | .000 |
| Completeheartblock | .000 |
| AtrialflutterwithvariableAVblockwithprematureventricula | .002 |
| Atrialsensedventricularpacedrhythm | .000 |
| AVdualpacedrhythm | .000 |
| prematureventricularoraberrantlyconductedcomplexes | .017 |
| Atrialpacedrhythm | .000 |
| UnusualPaxisandshortPRprobablejunctionaltachycardia | .000 |
| UnusualPaxisandshortPRprobablejunctionalrhythm | .000 |
| UnusualPaxisandshortPRprobablejunctionalbradycardia | .000 |
| PrematureventricularcomplexesandFusioncomplexes | .003 |
| occasionalPrematureventricularcomplexesandFusioncomplexes | .001 |
| consecutivePrematureventricularcomplexesandFusioncomplexes | .001 |
| occasionalandconsecutivePrematureventricularcomplexesandF | .000 |
| frequentPrematureventricularcomplexesandFusioncomplexes | .001 |
| frequentandconsecutivePrematureventricularcomplexesandFus | .001 |
| SinusrhythmwithPrematuresupraventricularcomplexesandPrema | .000 |
| Fusioncomplexes | .015 |
| ventricularescapecomplexes | .001 |
| occasionalandconsecutivePrematureventricularcomplexes | .004 |
| occasionalPrematureventricularcomplexes | .040 |
| frequentandconsecutivePrematureventricularcomplexes | .002 |
| frequentPrematureventricularcomplexes | .008 |
| consecutivePrematureventricularcomplexes | .006 |
| Supraventriculartachycardia | .000 |
| occasionalandconsecutive | .004 |
| occasional | .044 |
| frequentandconsecutive | .003 |
| frequent | .011 |
| consecutive | .006 |
| markedsinusarrhythmia | .013 |
| **COMPOSITE FEATURES (constructed using features from CIE statements):** | |
| PR_MEASURABLE (PR-value measurable) | .873 |
| Lateral_location (Any pathology laterally observed) | .358 |
| Anterior_location (Any pathology anteriorly observed) | .322 |
| Isolated_anterior_location (Any pathology observed only anteriorly) | .180 |
| Isolated_inferior_location(Any pathology observed only inferiorly) | .234 |
| Isolated_lateral_location (Any pathology observed only laterally) | .168 |
| Inferior_location (Any pathology observed inferiorly) | .266 |
| Myocardial_infarction (Any mention of myocardial infarction) | .340 |
| ST_segment_depression (Any mention of ST segment depression) | .245 |
| Myocardial_infarction_classified | .184/.081/.075 |
| T_wave_pathology (T wave pathology observed anywhere) | .449 |
| Septal_isolated_MI (Only septal myocardial infarction observed, any type) | .047 |
| Septal_MI (Septal myocardial infarction observed, any type) | .110 |
| Anterior_isolated_MI (Only anterior myocardial infarction observed, any type) | .143 |
| Anterior_MI (Any mention of anterior myocardial infarction) | .166 |
| Lateral_MI (Any mention of lateral myocardial infarction) | .042 |
| Lateral_isolated_MI (Any mention of only lateral myocardial infarction) | .018 |
| Inferior_MI (Any mention of inferior myocardial infarction) | .185 |
| Inferoporsterior_isolated_MI (Any mention of only infero-posterior myocardial infarction) | .184 |
| Posterior_isolated_MI (Any mention of only posterior myocardial infarction) | .014 |
| MI_classified (Myocardial infarction type classified) | .085/.010/.002/.015/.214/.007 |
| MI_classified_clinical (Myocardial infarction type classification simplified) | .299/.016 |
| Anterior_MI_classified (Anterior myocardial infarction type classification) | .019/.002/.001/.012/.127 .006 |
| Lateral_MI_classified (Lateral myocardial infarction type classification) | .010/.011/.001/.003/.016/.003 |
| Inferior_MI_classified (Inferiorn myocardial infarction type classification) | .068/.002/.002/.006/.106/.001 |
| ST_segement_depression_anterior_isolated (any ST segment depression observed only anteriorly) | .018 |
| ST_segement_depression_anterior (any ST segment depression observed anteriorly) | .102 |
| ST_segement_depression_anterolateral_isolated (any ST segment depression observed only in anterolateral area) | .084 |
| ST_segement_depression_lateral (any ST segment depression observed in lateral area) | .173 |
| ST_segement_depression_lateral_isolated (any ST segment depression observed only in lateral area) | .069 |
| ST_segement_depression_inferolateral (any ST segment depression observed in inferolateral area) | .089 |
| ST_segment_depression_inferolateral_isolated (any ST segment depression observed in only inferolateral area) | .020 |
| ST_segment_depression_inferior (any ST segment depression observed in inferior area) | .048 |
| ST_segment_depression_inferior_isolated (any ST segment depression observed only in inferior area) | .028 |
| ST_segment_depression_classified (any ST segment depression or marked ST segment depression observed) | .187/.020 |
| ST_segement_depression_anterior_classified (any ST segment depression or marked ST segment depression observed in anterior area) | .092/.011 |
| ST_segement_depression_lateral_classified (any ST segment depression or marked ST segment depression observed in lateral area) | .162/.011 |
| ST_segement_depression_inferolateral_classified (any ST segment depression or marked ST segment depression observed in inferolateral area) | .081/.008 |
| ST_segment_depression_inferior_classified (any ST segment depression or marked ST segment depression observed in inferior area) | .042/.006 |
| T_wave_pathology_anterior_isolated (any T wave pathology observed only in anterior area) | .034 |
| T_wave_pathology_anterior (any T wave pathology observed in anterior area) | .164 |
| T_wave_pathology_anterolateral_isolated (any T wave pathology observed in only anterolateral area) | .131 |
| T_wave_pathology_lateral (any T wave pathology observed in lateral area) | .305 |
| T_wave_pathology_lateral_isolated (any T wave pathology observed in only lateral area) | .144 |
| T_wave_pathology_inferolateral (any T wave pathology observed in inferolateral area) | .174 |
| T_wave_pathology_inferolateral_isolated (any T wave pathology observed in only inferolateral area) | .030 |
| T_wave_pathology_inferior (any T wave pathology observed in inferior area) | .075 |
| T_wave_pathology_inferior_isolated (any T wave pathology observed in only inferior area) | .045 |
| T_wave_pathology_classified (T wave pathology or marked T wave pathology observed anywhere) | .314/.055 |
| T_wave_pathology_anterior_isolated_classified (T wave pathology or marked T wave pathology observed only in anterior area) | .033/.001 |
| T_wave_pathology_anterior_classified (T wave pathology or marked T wave pathology observed in anterior area) | .111/.053 |
| T_wave_pathology_lateral_classified (T wave pathology or marked T wave pathology observed in lateral area) | .251/.054 |
| T_wave_pathology_lateral_isolated_classified (T wave pathology or marked T wave pathology observed in only lateral area) | .143/.001 |
| T_wave_pathology_inferolateral_classified (T wave pathology or marked T wave pathology observed in inferolateral area) | .172/.001 |
| T_wave_pathology_inferolateral_isolated_classified (T wave pathology or marked T wave pathology observed only in inferolateral area) | .030 |
| T_wave_pathology_inferior_classified (T wave pathology, or marked pathology, any mention) | .075/.001 |
| T_wave_pathology_inferior_isolated_classified (inferior region T wave pathology, or marked pathology) | .045/.001 |
| Marked_ST_abnormality (Marked ST abnormality, any mention) | .020 |
| Sinus_rhythm (Sinus rhythm, any mention) | .413 |
| PVC (Premature Ventricual Contraction, any mention) | .084 |
| LVH_wide (Left Ventricular Hypetrophy, definite and probable combined) | .077 |
| NIVCD (Non-Specific Intraventricular Conduction Block/Disorder/Delay | .018 |

**Supplementary Section 2. Python (version 3.10.12) code used in analysis**

import pandas as pd

import numpy as np

from sklearn.model_selection import train_test_split

from sklearn.metrics import roc_auc_score, roc_curve

from sklearn.inspection import permutation_importance

from sklearn.linear_model import LogisticRegression

from sklearn.tree import DecisionTreeClassifier

from sklearn.ensemble import RandomForestClassifier

from sklearn.svm import SVC

from sklearn.neighbors import KNeighborsClassifier

import matplotlib.pyplot as plt

from sklearn.ensemble import GradientBoostingClassifier

from sklearn.naive_bayes import GaussianNB

from sklearn.ensemble import AdaBoostClassifier

from sklearn.neural_network import MLPClassifier

from sklearn.model_selection import StratifiedKFold, cross_val_score

from joblib import Parallel, delayed

from tqdm.auto import tqdm

from sklearn.base import clone

from tqdm.contrib.concurrent import process_map

from functools import partial

from sklearn.model_selection import GridSearchCV

from sklearn.metrics import confusion_matrix

def calculate_sensitivity_specificity(y_test, y_pred_proba, threshold):

# Calculate binary predictions with threshold

y_pred = (y_pred_proba >= threshold).astype(int)

# Calculate confusion matrix

tn, fp, fn, tp = confusion_matrix(y_test, y_pred).ravel()

# Calculate sensitivity and specificity

sensitivity = tp / (tp + fn)

specificity = tn / (tn + fp)

return sensitivity, specificity

def get_feature_importances(model, X_test, y_test, n_top_importances, feature_names, metric='roc_auc'):

# Calculate permutation importances

result = permutation_importance(model, X_test, y_test, scoring=metric, n_repeats=10, n_jobs=1, random_state=42)

# Create a DataFrame with feature importances and feature names

feature_importances = pd.DataFrame({'feature': feature_names, 'importance': result.importances_mean})

# Sort the DataFrame by importance in descending order

feature_importances = feature_importances.sort_values(by='importance', ascending=False)

# Return the top n feature importances

return feature_importances.head(n_top_importances)

def process_model_wrapper(args):

model_class, param_grid, X_train, y_train, X_test, y_test, n_top_importances, risk_levels = args

return process_model(model_class, param_grid, X_train, y_train, X_test, y_test, n_top_importances, risk_levels)

def process_model(model_constructor, param_grid, X_train, y_train, X_test, y_test, n_top_importances, risk_levels):

# Perform hyperparameter optimization using GridSearchCV

grid_search = GridSearchCV(model_constructor(), param_grid, scoring='roc_auc', n_jobs=1, cv=10)

grid_search.fit(X_train, y_train)

best_model = grid_search.best_estimator_

# Print the best model parameters

print(f"Best parameters for {best_model.__class__.__name__}: {grid_search.best_params_}")

# Train and evaluate the model

best_model.fit(X_train, y_train)

y_pred_proba = best_model.predict_proba(X_test)[:, 1]

auroc = roc_auc_score(y_test, y_pred_proba)

importances = get_feature_importances(best_model, X_test, y_test, n_top_importances, X_train.columns)

model_results = {

'name': best_model.__class__.__name__,

'auroc': auroc,

'importances': importances,

'y_pred_proba': y_pred_proba,

'sensitivities': [],

'specificities': []

}

# Calculate sensitivity and specificity at each risk level

for risk_level in risk_levels:

sensitivity, specificity = calculate_sensitivity_specificity(y_test, y_pred_proba, risk_level)

model_results['sensitivities'].append(sensitivity)

model_results['specificities'].append(specificity)

return model_results

def train_and_evaluate_models_parallel(train_df, test_df, target_col, n_top_importances, risk_levels):

# Preparing the data

X_train = train_df.drop(target_col, axis=1)

y_train = train_df[target_col]

X_test = test_df.drop(target_col, axis=1)

y_test = test_df[target_col]

# Initializing the models with their parameter grids

models = [

(LogisticRegression, {'penalty': ['l1', 'l2'], 'solver': ['liblinear'], 'C': [0.01, 0.1, 1.0], 'max_iter': [200]}),

(RandomForestClassifier, {'n_estimators': [10, 50, 100, 200], 'criterion': ['gini', 'entropy'], 'max_depth': [None, 10, 20, 30], 'min_samples_split': [2, 5, 10]}),

]

results = []

feature_importances = []

# Prepare the ROC plot

plt.figure(figsize=(12, 6))

plt.subplot(1, 2, 1)

process_model_partial = lambda model_class, param_grid: process_model(model_class, param_grid, X_train, y_train, X_test, y_test, n_top_importances, risk_levels)

model_results = process_map(process_model_wrapper, [(mc, pg, X_train, y_train, X_test, y_test, n_top_importances, risk_levels) for mc, pg in models], max_workers=len(models), chunksize=1)

for model_result in model_results:

name = model_result['name']

auroc = model_result['auroc']

importances = model_result['importances']

y_pred_proba = model_result['y_pred_proba'] # Assuming the model results dictionary contains 'y_pred_proba'

# Calculate ROC curve

fpr, tpr, _ = roc_curve(y_test, y_pred_proba)

# Plot ROC curve

plt.plot(fpr, tpr, label=f'{name} (AUROC = {auroc:.4f})')

results.append((name, auroc, model_result['sensitivities'], model_result['specificities']))

feature_importances.append((name, importances))

# Customize the ROC plot

plt.plot([0, 1], [0, 1], 'k--', label='Random (AUROC = 0.5)')

plt.xlabel('False Positive Rate')

plt.ylabel('True Positive Rate')

plt.title('Receiver Operating Characteristic (ROC) Curves')

plt.legend(loc='lower right')

plt.grid()

# Prepare the importance plot

plt.subplot(1, 2, 2)

width = 0.10

ind = np.arange(n_top_importances)

total_models = len(models)

for i, (name, importances) in enumerate(feature_importances):

plt.bar(ind + i * width, importances['importance'], width, label=name)

# Space out xticks based on the number of models

xticks_position = ind + width * (total_models - 1) / 2

plt.xlabel('Features')

plt.ylabel('Importance')

plt.title('Top Feature Importances')

plt.xticks(xticks_position, importances['feature'], rotation=45) # Rotate x-labels by 45 degrees

plt.legend(loc='upper right')

plt.grid()

# Show the plots

plt.tight_layout()

plt.show()

return results, feature_importances

df = df.dropna()

train_df = df[df.TRAINING_VALIDATION_SPLIT == 1].drop(["TRAINING_VALIDATION_SPLIT", "id", "supertunniste"], axis=1)

test_df = df[df.TRAINING_VALIDATION_SPLIT == 2].drop(["TRAINING_VALIDATION_SPLIT", "id", "supertunniste"], axis=1)

target_col = "SCDv5_5V_USE_THIS_FOR_MODEL_DEVELOPMENT"

n_top_importances = 10

print("Training patients count by target class: \n{}".format(train_df[target_col].value_counts()))

print("\nTesting patients count by target class: \n{}".format(test_df[target_col].value_counts()))

risk_levels = [0.05, 0.10]

results, feature_importances = train_and_evaluate_models_parallel(train_df, test_df, target_col, n_top_importances, risk_levels)

for (name, auroc, sensitivities, specificities), (_, importances) in zip(results, feature_importances):

print(f"\nModel: {name}")

# Print AUROC results

print("\nAUROC Result:")

print(f"Area Under the Receiver Operating Characteristic Curve (AUROC): {auroc:.4f}")

# Print feature importances

print("\nTop Feature Importances:")

print(importances.to_string(index=False))

# Print sensitivity and specificity at risk levels

print("\nSensitivity and Specificity at Risk Levels:")

for i, risk_level in enumerate(risk_levels):

print(f"Sensitivity at risk level {risk_level}: {sensitivities[i]}")

print(f"Specificity at risk level {risk_level}: {specificities[i]}")

print("-"*80) # separator for clarity

**Supplementary Section 2. R-code used in analysis**

library(caret)
library(xgboost)
library(ParBayesianOptimization)

#### Make this example reproducible and make train and test sets for XGBoost ##

set.seed(617)

dat_xgb<-dat[,vars_x] # select Xs to a data frame

dat_xgb$Y<-dat$endpoint # add Y to the data frame

#split into training (70%) and testing set (30%)

parts<-createDataPartition(dat_xgb$Y, p = .7, list = F)

train<-dat_xgb[parts, ]

test<-dat_xgb[-parts, ]

#define predictor and response variables in training and test d test sets

y_no<-which(names(dat_xgb)=='Y')

train_x = data.matrix(train[, -y_no])

train_y = train[,y_no]

test_x = data.matrix(test[, -y_no])

test_y = test[,y_no]

#define final training and testing sets

xgb_train = xgb.DMatrix(data = train_x, label = train_y)

xgb_test = xgb.DMatrix(data = test_x, label = test_y)

######BAYES OPT hyperpar search ##

scoring_function <- function (eta, gamma, max_depth, min_child_weight, subsample, max_delta_step) {

dtrain <- xgb.DMatrix(data = train_x, label = train_y)

pars <- list(

eta = eta,

gamma = gamma,

max_depth = max_depth,

min_child_weight = min_child_weight,

subsample = subsample,

max_delta_step = max_delta_step,

booster = "gbtree",

objective = "binary:logistic",

eval_metric = "auc",

verbosity = 0

)

xgbcv <- xgb.cv(

params = pars,

scale_pos_weight = 30, # optional

nthread =6,

data = dtrain,

nfold =10,

nrounds = 75,

prediction = TRUE,

showsd = TRUE,

early_stopping_rounds = 20,

maximize = T,

stratified = TRUE

)

return(

list(

Score = max(xgbcv$evaluation_log$test_auc_mean),

nrounds = xgbcv$best_iteration

)

)

}

bounds <- list(

eta = c(0, 1),

gamma =c(0, 10),

max_depth = c(1L, 15L),

min_child_weight = c(2, 25),

subsample = c(0.1, 1),

max_delta_step=c(0,10)

)

opt_obj <- bayesOpt(FUN = scoring_function, bounds = bounds,initPoints = 7, iters.n =500, iters.k=5,verbose=2,)

opt_obj$scoreSummary

getBestPars(opt_obj)

## Save the best hyperparams ###

tuned_params <- list(eta = getBestPars(opt_obj)[1],

gamma = getBestPars(opt_obj)[2],

max_depth = getBestPars(opt_obj)[3],

min_child_weight = getBestPars(opt_obj)[4],

subsample = getBestPars(opt_obj)[5],

max_delta_step = getBestPars(opt_obj)[6],

#scale_pos_weight = 45, # negative / postive cases

objective = "binary:logistic")

numrounds <- opt_obj$scoreSummary$nrounds[

which(opt_obj$scoreSummary$Score

== max(opt_obj$scoreSummary$Score))]

### Fit the Final model with opitmized hyperparams ###

fit_tuned <- xgboost(params = tuned_params,

data = train_x,

label = train_y,

nrounds = numrounds,

eval_metric = "auc")

y_pred_tuned <- predict(fit_tuned, test_x)

# continue with ‘y_pred_tuned / test_y’ to obtain which ever descri
